# Short-term results of early switch from Ranibizumab to Aflibercept in poor or non responder age related macular degeneration in clinical practice

**DOI:** 10.1186/s40942-020-00212-5

**Published:** 2020-05-14

**Authors:** Luciana de Sá Quirino-Makarczyk, Maria de Fátima Sainz Ugarte, Bruna Viana Vieira, Sérgio Kniggendorf, Caio Vinicius Saito Regatieri

**Affiliations:** 1 Department of Retina and Vitreous of Hospital Oftalmológico de Brasília, Brasília, Brazil; 2 Hospital Oftalmológico de Brasília, Brasília, Brazil; 3 Federal University of São Paulo (UNIFESP), São Paulo, Brazil

**Keywords:** Aflibercept, Ranibizumab, Age-related macular degeneration, Vascular endothelial growth factor

## Abstract

**Background:**

To evaluate the change in best corrected visual acuity (VA) and central macular thickness (CMT) following treatment with intravitreal aflibercept (AFL) in patients poorly responders or non responders to ranibizumab (RBZ).

**Methods:**

Charts of patients injected with RBZ from January 2016 to December 2018 (548 cases) due to neovascular age-related macular degeneration (nAMD) were reviewed. Fifty-six cases met our criteria for poor responders to RBZ (CMT decreased between 5 and 15% over treatment) or for non responders to RBZ (CMT decreased less than 5% or increased over treatment).

**Results:**

After the third AFL injection, CMT decreased from 384.38 ± 123.20 μm to 296.18 ± 70.52 μm in the non-responder group and from 320.00 ± 82.05 μm to 282.27 ± 56.86 μm in the poor responder group. Although decrease in macular thickness was overall achieved 3 months after switching to AFL, it was not translated in VA improvement.

**Conclusions:**

it was observed that nAMD patients classified as RBZ non-responders tend to respond better to AFL than RBZ poor-responders anatomically, without correspondent improvement in VA.

## Introduction

Age-related macular degeneration (AMD) is a degenerative pathology that can cause severe and permanent vision loss. The neovascular AMD is characterized by growth of abnormal vessels that lead to leakage of sub and intraretinal fluid. The growth of those abnormal vessels is mainly caused by an increase in the production of angiogenic factors, being the vascular endothelial growth factors (VEGF) the most important. VEGF activates angiogenesis and vascular permeability [1, 2].

Vascular endothelial growth factors inhibitors have been used for neovascular AMD treatment. The most commonly used anti-VEGF factors in the clinical practice nowadays are: ranibizumab (Lucentis^®^, Novartis), aflibercept (Eylea^®^, Bayer) and bevacizumab (Avastin^®^, Roche Pharma), although the use of the last one is off-label [2, 3].

Ranibizumab (RBZ) is a recombinant humanized IgG1 monoclonal antibody fragment (48 kDa), that neutralizes all active forms of VEGF-A. Aflibercept (AFL) is a fusion protein (115 kDa) that neutralizes all forms of VEGF-A, VEGF-B and placental growth factor 1 and 2. In vitro studies showed that AFL has a longer half-life than ranibizumab [4]. Regarding to the affinity of these drugs to VEGF, studies showed some conflicting results [5].

The safety and efficacy of RBZ as intravitreal injection for neovascular AMD (nAMD) was shown in several multicentric studies [6, 7], as monthly or pro re nata protocols (PRN) [8]. Although some patients achieved positive results with the treatment, several others continued to experience progressive visual loss and macular fluid even with monthly intravitreal injections. The first and second years of CATT study showed that more than 50% of patients that received RBZ had macular fluid persistence after the twelfth month period of analysis [9, 10]. Those patients can be defined as resistant to treatment. Although the resistance mechanisms are not explained yet, tolerance and tachyphylaxis to RBZ are the possible causes [11]. The HARBOR study showed that higher doses of RBZ were not superior if compared to the 0.5 mg dose and that monthly administration leads to superior results than PRN protocol [12]. Contradicting HARBOR study, SAVE study showed that loss of efficacy can be resolved, in some patients, using higher doses [13].

The studies VIEW 1 and VIEW 2 compared the efficacy and safety of monthly intravitreal injections of RBZ and AFL and concluded that they were equally efficient to improve and prevent vision loss. Although the group treated with AFL needed an average of five less injections after 96 weeks [14].

Recently, several studies discussed the development of anti-VEGF resistance and have demonstrated that the switch of intravitreal drug can be effective in treatment [15–18].

Our goal in this study was to compare visual and macular results over the first 3 months of treatment after early switch to aflibercept in the treatment of neovascular AMD with complete or partial resistance to RBZ.

Our secondary goal was to analyze if the poor responders and non responders groups differ in tomographic macular findings when injected with AFL.

## Methods

This study was approved by Research Ethics Committee of *Hospital Oftalmológico de Brasília* and it was performed in accordance to the Declaration of Helsinki that sets out rules on research involving humans.

We retrospectively evaluated patients with nAMD that were injected with RBZ and posteriorly treated with AFL between January 2016 to December 2018. Patients identified as poor or non-responders to RBZ treatment were selected for more detailed analysis.

All charts selected for analysis were from patients who received 0.5 mg of intravitreal RBZ for 3 months before early switch to AFL. Also, after drug switch, all selected patients had to meet the criteria of at least 3 monthly loading doses of AFL 2 mg.

Patients were imaged using the Heidelberg Spectralis (Heidelberg Engineering, Germany^®^) in spectral domain-optical coherence tomography (OCT) mode. Automatic quantitative maps of retinal thickness were evaluated: a baseline map and a map 1 month after each injection.

Patients included were considered, according to OCT findings, either: (1) poor-responsive, whose central macular thickness minimally changed (5–15%) over treatment with RBZ or (2) non-responsive, whose central macular thickness did not change (< 5%) or increased over treatment with RBZ.

The included patients should be pseudophakic, with visual acuity better than 1.3 logMAR (visual acuity scale). In cases with bilateral pathology, both eyes could be included in the study if they met the inclusion criteria. The interval between the last RBZ injection and the first AFL injection should be superior to 4 and inferior to 6 weeks.

The exclusion criteria were: patients with macular epiretinal membranes, glaucoma, neovascular subretinal membranes from another etiology, foveal fibrosis or retinal pigment epithelium (RPE) tears. In none of the patients included in this study RPE detachments were a parameter to start treatment.

Collected data from patients included in this study were age, gender, best corrected vision acuity (VA), OCT morphology and central macular thickness (CMT), complications and adverse events associated. VA was measured with Snellen chart, and the decimal visual acuity was converted to the logarithm of the minimal angle of resolution (logMAR) units for the statistical analysis. Baseline mean VA and CMT were compared with their outcomes one month after each injection. Comparisons of mean VA and CMT changes between treatments after ranibizumab and aflibercept injections were done. Data are presented as mean and standard deviations.

Distribution of data for normality was checked using Shapiro–Wilk test. The significance of any difference in means was evaluated by nonparametric test (Mann–Whitney) and for parametric analysis the T-test was used. Statistical significance was defined as p < 0.05.

## Results

Five hundred forty-eight charts of patients treated with intravitreal injections during January 2016 to December 2018 were reviewed. Fifty-six patients met the inclusion criteria. Among those, 11 were considered poor responders and 45 were considered non responders to RBZ treatment.

The average age was 70.3 years old (ranged from 53 to 93 years old). Sixty-six per cent (66.07%) were female and thirty-three per cent (33.09%) were male. Before switching to AFL, the VA ranged from 0.1 to 1.3 logMAR.

**Fig. 1:**
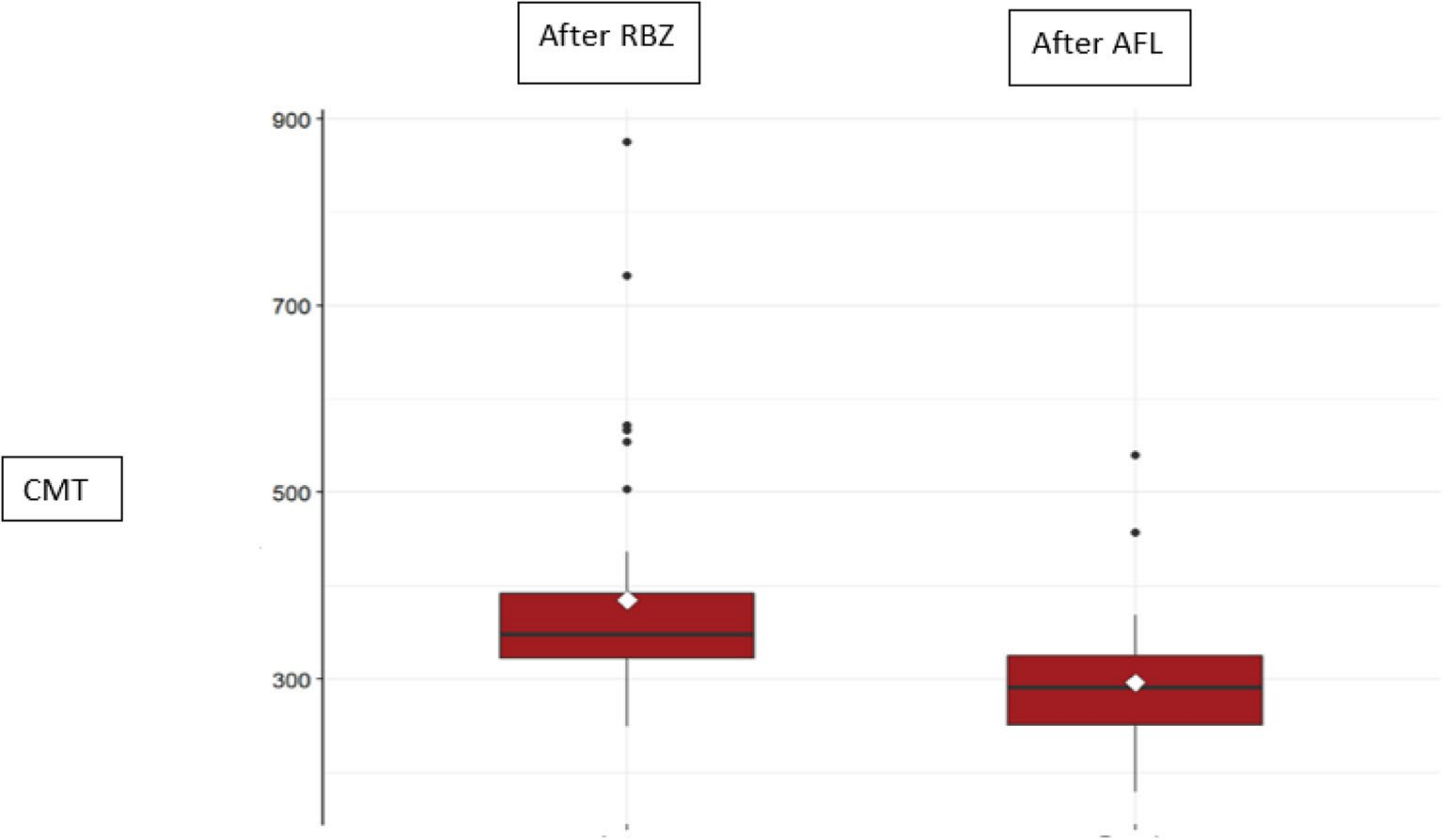
Central macular thickness (CMT) of the ranibizumab (RBZ) non-responder patients after the 3rd injection with RBZ (mean: 384.38 ± 123.20 μm) and after the 3rd injection with aflibercept (AFL) (mean: 296.18 ± 70.52 μm)

Figure 1 shows box plots comparing mean central macular thickness of the ranibizumab non-responder patients after the third injection of ranibizumab and then after the third injection of aflibercept.

**Fig. 2:**
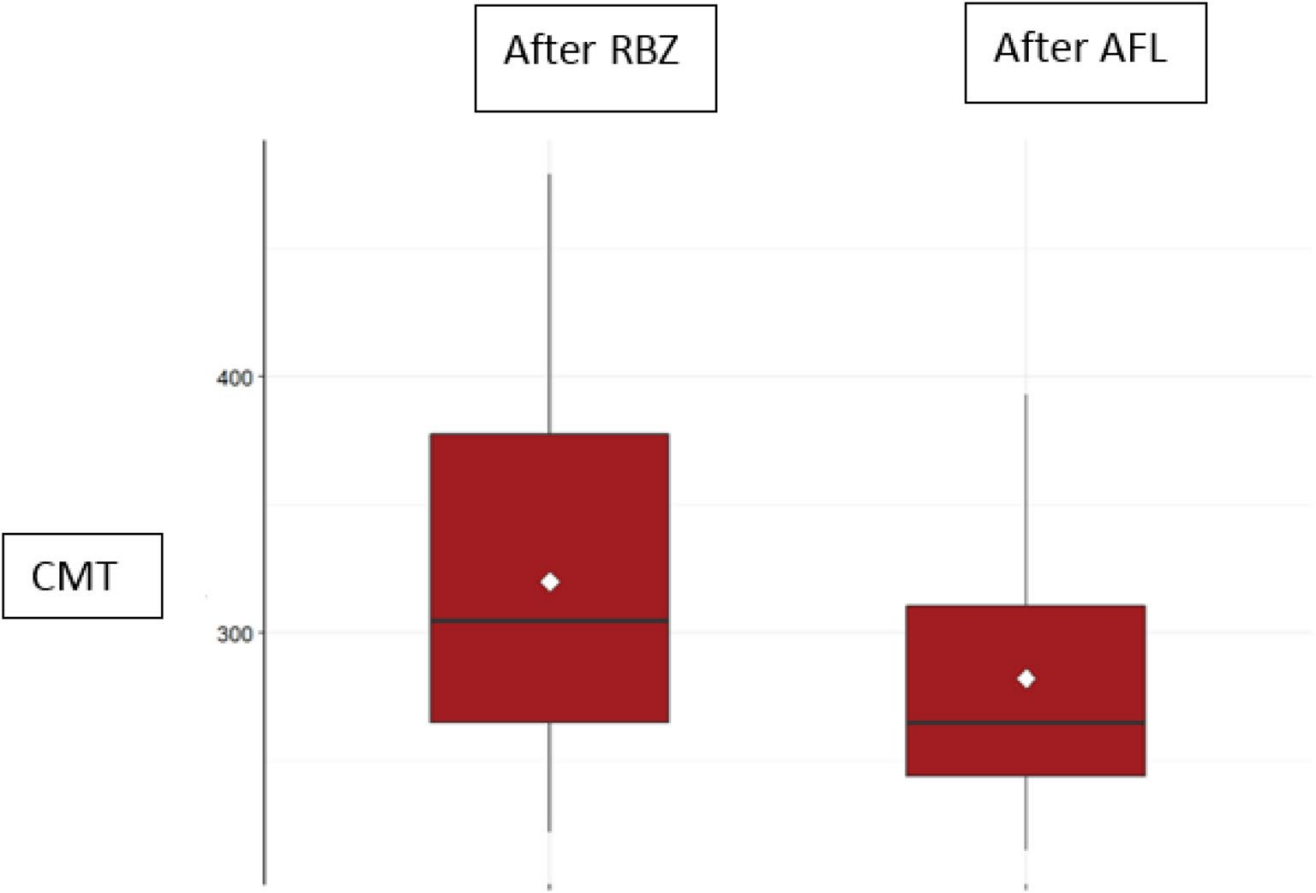
Central macular thickness (CMT) of the ranibizumab (RBZ) poor-responder patients after the 3rd injection with RBZ (mean: 320.00 ± 82.05 μm) and after the 3rd injection with aflibercept (AFL) (mean: 282.27 ± 56.86 μm)

Figure 2 shows box plot comparing mean CMT of the RBZ poor-responder group after the third injection of RBZ and then after the third injection of AFL.

The Mann–Whitney test was used to compare final CMT after the two treatments. In both groups, the CMT decreased significantly after last injection of AFL as compared to the values measured 1 month after the last RBZ injection (p ≤ 0.05). However, this difference was more significant in the group of non-responders (p-value < 0.01) than in the group of poor responders (p-value = 0.01247).

**Fig. 3:**
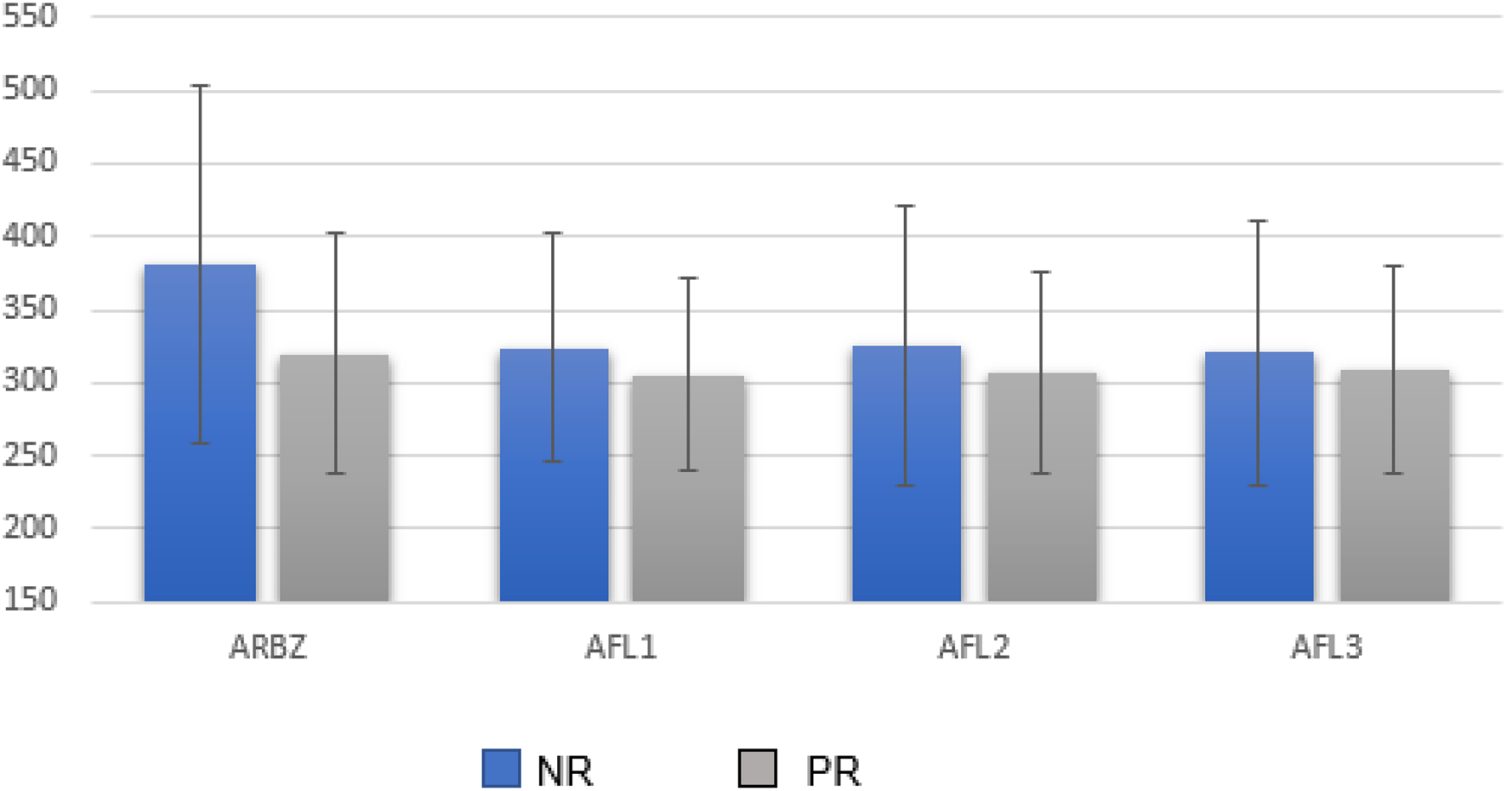
Central macular thickness (CMT) monthly variation, measured in μm, after the third dose of ranibizumab (ARBZ), after the first dose of Aflibercept (AFL1), after the second dose of aflibercept (AFL2), after the third dose of aflibercept (AFL3); *NR* non-responder, *PR* poor responder

Analysis of the CMT monthly variation (baseline and 1 month after each injection) using Wilcoxon test showed that the results obtained are mainly due to the first injection of AFL in both groups (p ≤ 0.05). The CMT monthly variation, Fig. 3, confirmed that the non responder group had a better response than the poor responder group and that this difference was evident after the first dose of AFL (p = 0.00000045 for the non responder group and p = 0.0021 for the poor responder group). The second and third injections of AFL did not induce additional statistically significant differences in CMT.

**Fig. 4:**
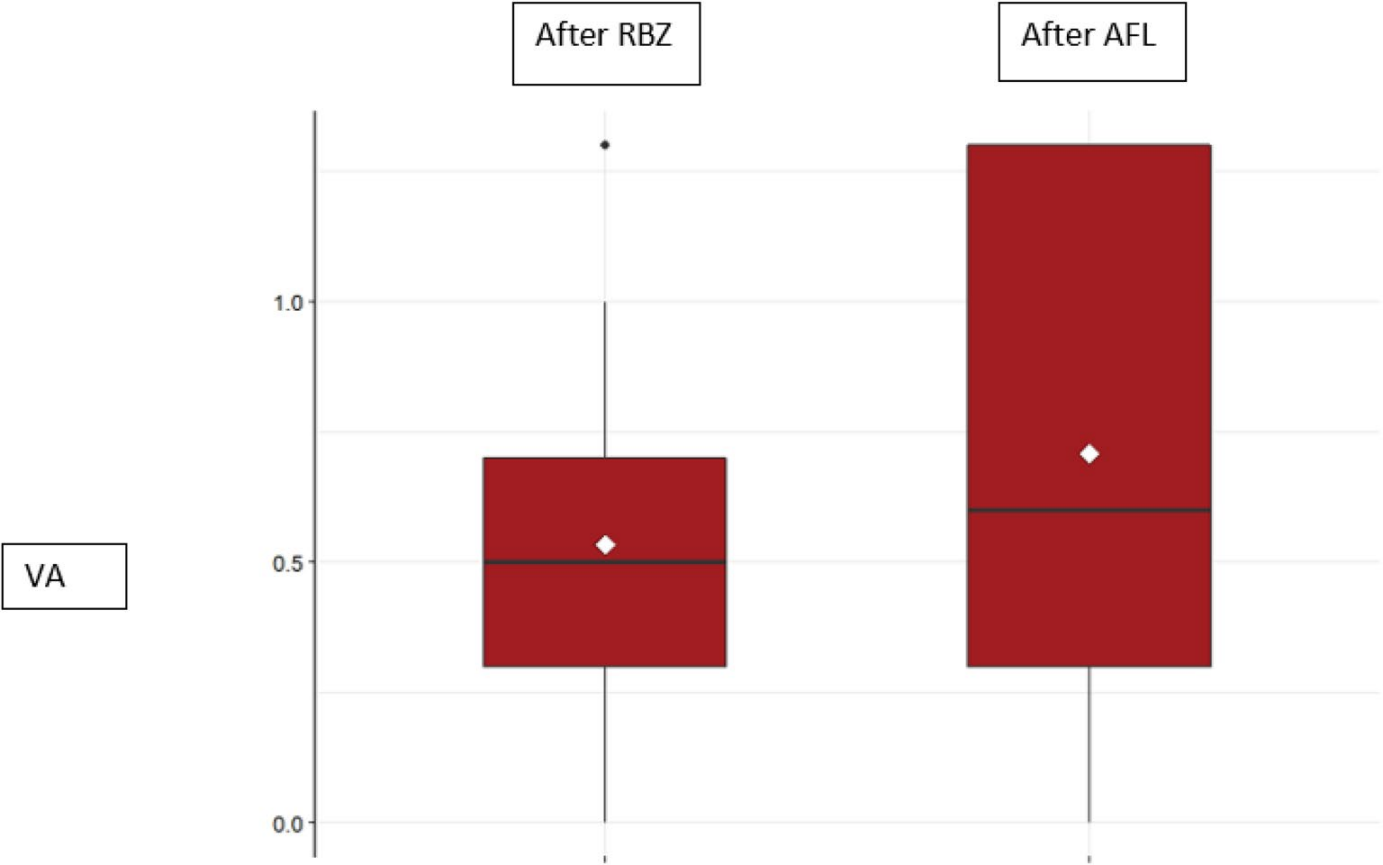
Visual acuity (VA) of the ranibizumab (RBZ) non-responder patients after the 3rd injection of RBZ (mean: 0.53 ± 0.36 logMAR) and after the 3rd injection of aflibercept (AFL) (mean: 0.71 ± 0.44 logMAR)

**Fig. 5:**
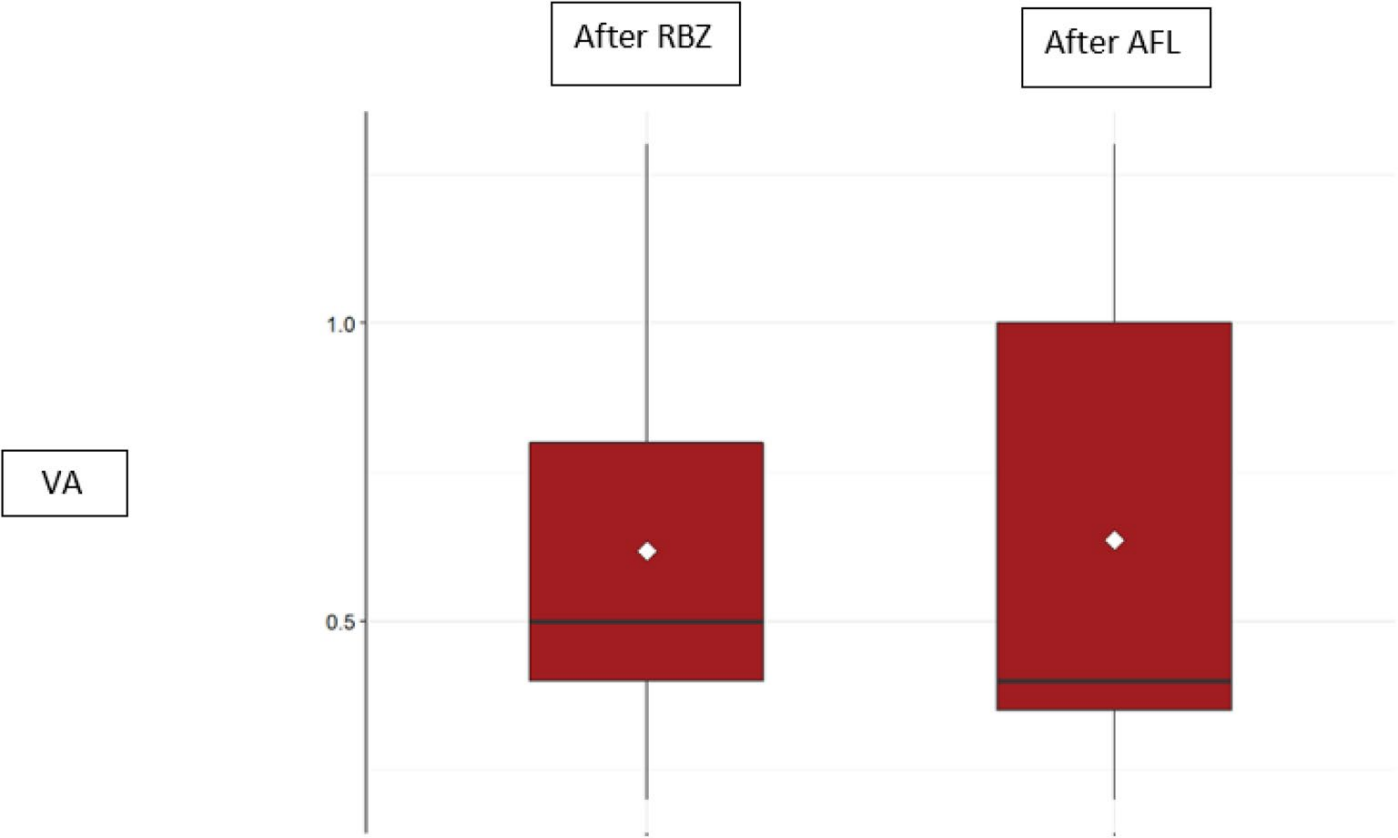
Visual acuity (VA) of the ranibizumab (RBZ) poor responder patients after the 3rd injection of RBZ (mean: 0.62 ± 0.33 logMAR) and after the 3rd injection of aflibercept (AFL) (mean: 0.64 ± 0.44 logMAR)

There was no improvement observed in VA (measured in logMAR) after third injection of AFL when compared to the last VA after RBZ treatment neither in the non-responder group (Fig. 4) nor in the poor responder group (Fig. 5).

The total of patients, poor or non-responders to RBZ, were divided after treatment with AFL in groups of poor responders, non responders and responders to AFL. In order to compare proportions between these patients, a two-way table of categorical variables was created (Table 1). According to this table, most of the RBZ non-responder patients, responded to AFL (48.9%). On the other hand, most of the RBZ poor-responder patients, did not respond to AFL (54.5%).

**Table 1:** A two-way table is used to compare proportions of the final number of poor responders, non responders and responders after treatment with aflibercept (AFL) in the ranibizumab (RBZ) non responder and poor responder groups

	AFL non-responder group	AFL poor-responder group	AFL responder group	Total
RBZ non-responder group	9	14	22	45
RBZ poor-responder group	6	2	3	11
Total	15	16	25	56

From the baseline OCT, it was observed that all the patients had disorganization of the outer retinal layers, including ellipsoid layer disruption, except by one patient of the non responder group. Additionally, 9 of the 11 patients of the poor responder group and 39 of the 45 patients from the non responder group had large neovascular membranes (≥ 4 disc areas). Based on OCT and fluorescein angiogram characteristics, in the group of non responders: 55.11% of membranes (23 eyes) were mixed type and 55.55% (24 eyes) were type 2. While in the group of poor responders, 54.54% of membranes (6 eyes) were mixed type and 45.45% (5 eyes) were type 2. No signs of chronicity such as outer retinal tubulations where found in any of the groups.

In all 548 patients injected from January 2016 to December 2018, no adverse effects or complications were observed, such as endophthalmitis, retinal detachments, uveitis or increase in intraocular pressure. Also, there were no systemic adverse events.

**Fig. 6:**
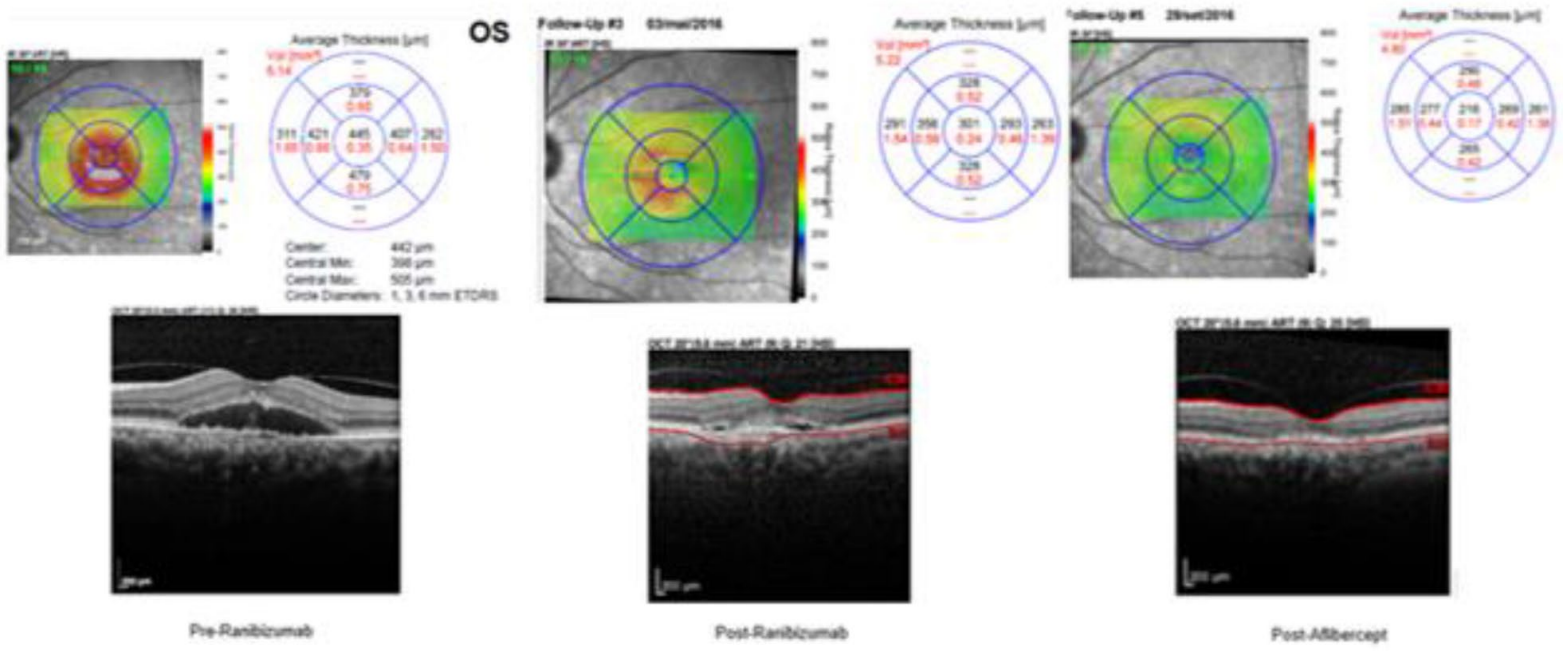
OCT 1: Pre-RBZ, CMT of 445 μm; OCT 2: Post-RBZ: persistence of a small amount of fluid despite 3 monthly injections of RBZ, CMT of 301 μm; OCT 3: resolution of persistent fluid after switching to AFL, CMT of 216 μm. *OCT* ocular coherence tomography, *RBZ* ranibizumab, *AFL* aflibercept; *CMT* central macular thickness

Figure 6 shows a series of OCT-Scan images of a patient who partially responded to RBZ and, after the switch to AFL, responded completely.

## Discussion

This study showed CMT improvement after treatment with intravitreal AFL in patients that were poor or non responders to RBZ, as found in other studies [19, 20]. However, the group of non responder patients was proven to have a better response to AFL than the group of poor responder patients. Visual acuity did not improve neither in the non-responsive nor in the poor responsive group after the third injection of AFL when compared to the last visit after treatment with 3 doses of RBZ.

The decision of switching anti-VEGF agents occurred on the follow up after the third injection of RBZ, which was proven not to be fully efficient. The early decision of switching anti-VEGF agents probably diminishes the visual harmful effects of the prolonged structural macular changes, allowing the patients to achieve their best potential vision. It was demonstrated that visual gain in the first 12 weeks treating macular edema was correlated to better final visual results, while the rapid improvement of CMT did not show to be predictive of better long term visual acuity [21]. Several studies did not demonstrate harmful effects to photoreceptors due to long term VEGF blockage [22, 23]. However, it has been hypothesized whether the extension of this effect could damage photoreceptors [24].

In this study, the majority of choroidal neovascular membranes were large and mixed type on baseline OCT. The membrane characteristics play a role on treatment outcome. Type 1 [19] and large [20, 21] choroidal neovascular membranes were defined as bad predictors for treatment outcome [19] as well as for poor response at 12 weeks [20, 21].

The results of this study could indicate a scenario of mainly two distinct responses to RBZ: (1) poor responders, where a possible tachyphylaxis or tolerance to RBZ had occurred and, due to a cross-tolerance effect, also a tendency to respond incompletely to AFL; (2) non responders, where VEGF-A is not the main causing factor for the neovascular growth.

Tachyphylaxis can occur after an initial dose or after series of drug doses [22–25]. Since tachyphylaxis cannot be overcome by increasing dosage, switching to a similar drug with different properties is recommended. Another reason for the poor response would be drug tolerance that could be caused by an over expression of VEGF receptors [11], which would result in reduced anti-VEGF effects. In both circumstances, a partial response would happen with initial injections of RBZ, but the switch to AFL would improve the results. In the RBZ non responder group, an early switch to AFL would have been even more beneficial than in the poor responder group, rising the hypothesis that VEGF-B is more implicated in the pathophysiology.

In almost all the studies regarding nAMD treatment, the presence of fluid on OCT was as a marker of disease activity and, therefore, indicated the need of retreatment. It was found that intraretinal fluid has a worse impact on VA if compared to subretinal fluid, especially when the fluid affects the fovea [24–27]. It was hypothesized that the refractory intraretinal fluid was caused by non-VEGF-mediated mechanisms, such as apoptotic or necrotic cell death [24], and was associated to a higher risk of fibrosis and geographic atrophy [25].

Controversially, the presence of subfoveal subretinal fluid may be associated with better visual outcomes and vision maintenance in the long term [26–29], since it would not represent neovascular activity, but a residual anatomical space caused by the failure of the neurosensory retina to reattach to the retinal pigment epithelium. One of the possible reasons for this is that the outer retinal layers in patients presenting with subfoveal subretinal fluid are less disrupted than in patients with intraretinal fluid involving the fovea [30]. Additionally, subretinal fluid could have a protective effect against geographic atrophy [31].

## Conclusions

In conclusion, the early switch of anti-VEGF drugs in patients resistant to treatment with RBZ is important to achieve rapid resolution of fluids, especially intraretinal fluid. This would improve the visual outcomes before subsequent deleterious effects of chronicity start to play a major role on results. It could also diminish the burden and risks associated with more frequent intravitreal injections. The main limiting factors of this study were the retrospective design and lack of control group.

## Data Availability

The data that support the findings of this study are available from *Hospital Oftalmológico de Brasília* but restrictions apply to the availability of these data, which were used under license for the current study, and so are not publicly available. Data are however available from the authors upon reasonable request and with permission of *Hospital Oftalmológico de Brasília*.

## Abreviations

VA: Visual acuity
CMT: Central macular thickness
AFL: Aflibercept
RBZ: Ranibizumab
nAMD: Neovascular age-related macular degeneration
OCT: Optical coherence tomography
VEGF: Vascular endothelial growth factor
PRN: Pro re nata
